# Primary Breast Extranodal Marginal Zone Lymphoma: A Case Report and Brief Review of the Literature

**DOI:** 10.1002/ccr3.70435

**Published:** 2025-04-15

**Authors:** Jessica Hui, Amani Bashir, Fabiana Policeni, Su Kim Hsieh

**Affiliations:** ^1^ Department of Radiology University of Iowa Hospitals and Clinics Iowa City Iowa USA; ^2^ Department of Pathology University of Iowa Hospitals and Clinics Iowa City Iowa USA

**Keywords:** lymphoma, neoplasms, pathology, radiology

## Abstract

Primary breast MALT lymphoma is an extremely rare and indolent malignancy with favorable outcomes. This case report aims to report typical imaging and pathologic findings to improve understanding and awareness of the presentation, diagnosis, and management of this uncommon entity.

## 1 Introduction

Primary breast lymphomas (PBL) are uncommon, having been reported to represent up to 1% of all cases of non‐Hodgkin lymphoma [1, 2] and only 0.4%–1% of all breast malignancies [2, 3]. Mucosa‐associated lymphoid tissue (MALT) lymphoma is an extranodal B‐cell lymphoma and a subtype of marginal zone lymphoma (MZL), which is a classically indolent form of non‐Hodgkin's lymphoma (NHL) with an average age of presentation above 65 years of age, with a slight female predilection. MALT lymphomas are more commonly associated with mucosal sites such as the gastrointestinal tract, lungs, and salivary glands, with the stomach being the most frequent extranodal site. As such, primary involvement of the breast is extremely unusual, only accounting for up to 9% of primary breast lymphomas and therefore fewer than 0.1% of all breast malignancies [4]. The clinical presentation of primary breast MALT lymphoma can be subtle, often manifesting asymptomatically as a painless, palpable mass. Due to its rarity and nonspecific presentation on mammography, this entity may not be considered in the initial differential, potentially leading to delays in diagnosis.

## 2 Case History

**FIGURE 1:**
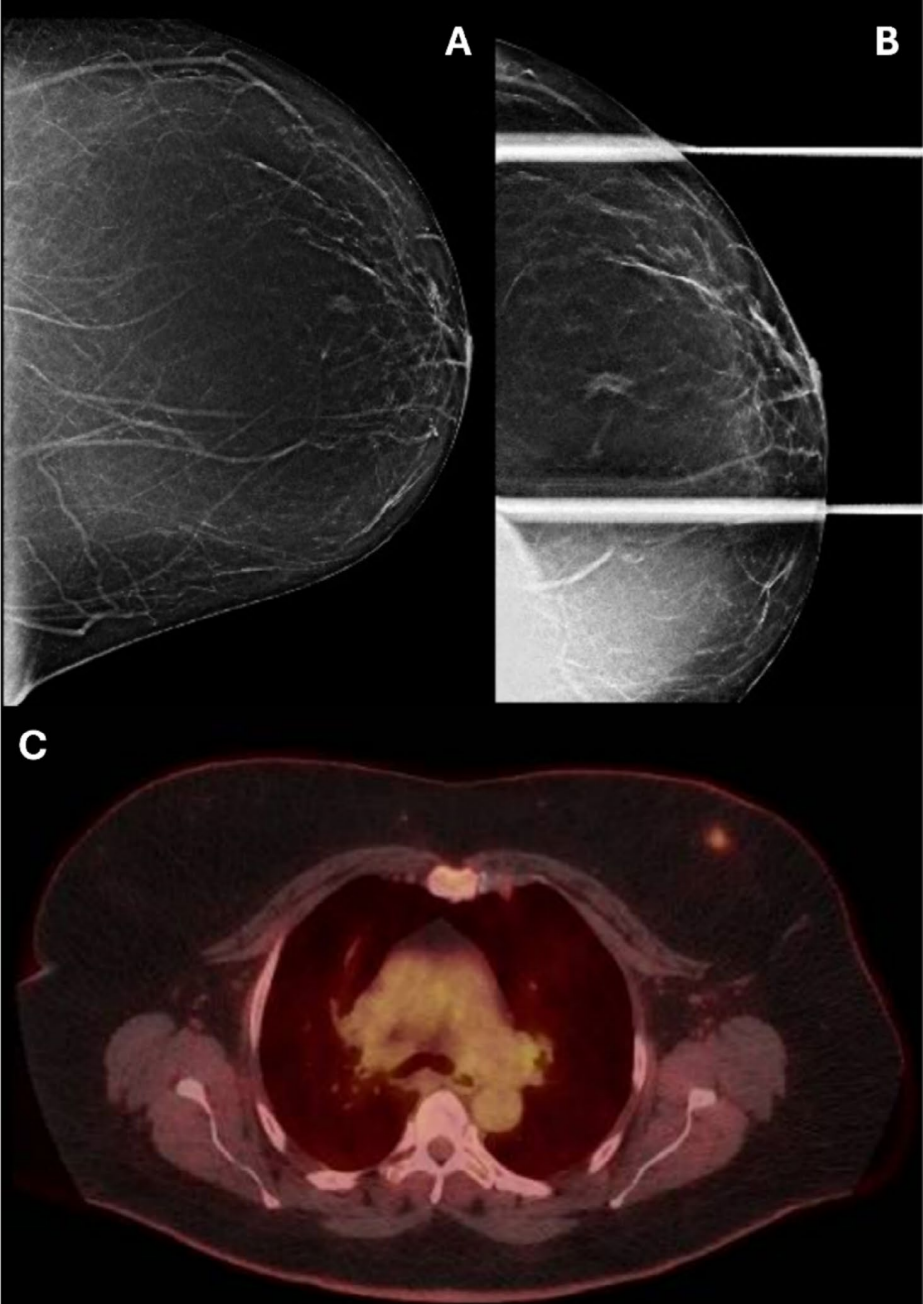
(A) Left breast CC view screening mammogram demonstrating an asymmetry in the anterior lateral left breast in the 12–1 o'clock position. (B) Spot compression CC view diagnostic mammogram of the left breast demonstrates that the asymmetry persists despite compression. Ultrasound (not shown) revealed no corresponding suspicious sonographic abnormality. (C) Subsequent post‐biopsy 18‐FDG PET‐CT demonstrating FDG avidity in the known mass with an SUVmax of 3.1 with uptake above that of the mediastinum but below that of the liver, consistent with Deauville 3.

The patient is a 65‐year‐old female with no significant past medical history who presented to her primary care physician for routine healthcare maintenance. No concerning breast symptoms were identified, with the patient denying breast pain, nipple discharge, or a palpable mass. She had no personal history of breast or ovarian cancer, no family history of breast cancer, with a low lifetime Tyrer‐Cuzick score of 12%. Given her age, she underwent routine screening mammography, with identification of a new asymmetry in the anterior lateral left breast. This was categorized as BI‐RADS 0, and a callback for diagnostic mammography was performed. On diagnostic mammogram, the asymmetry persisted on spot compression views (Figure 1A,B), although no sonographic correlate was definitively identified. This was classified as BI‐RADS 4, with a recommendation for tissue sampling.

## 3 Methods

**FIGURE 2:**
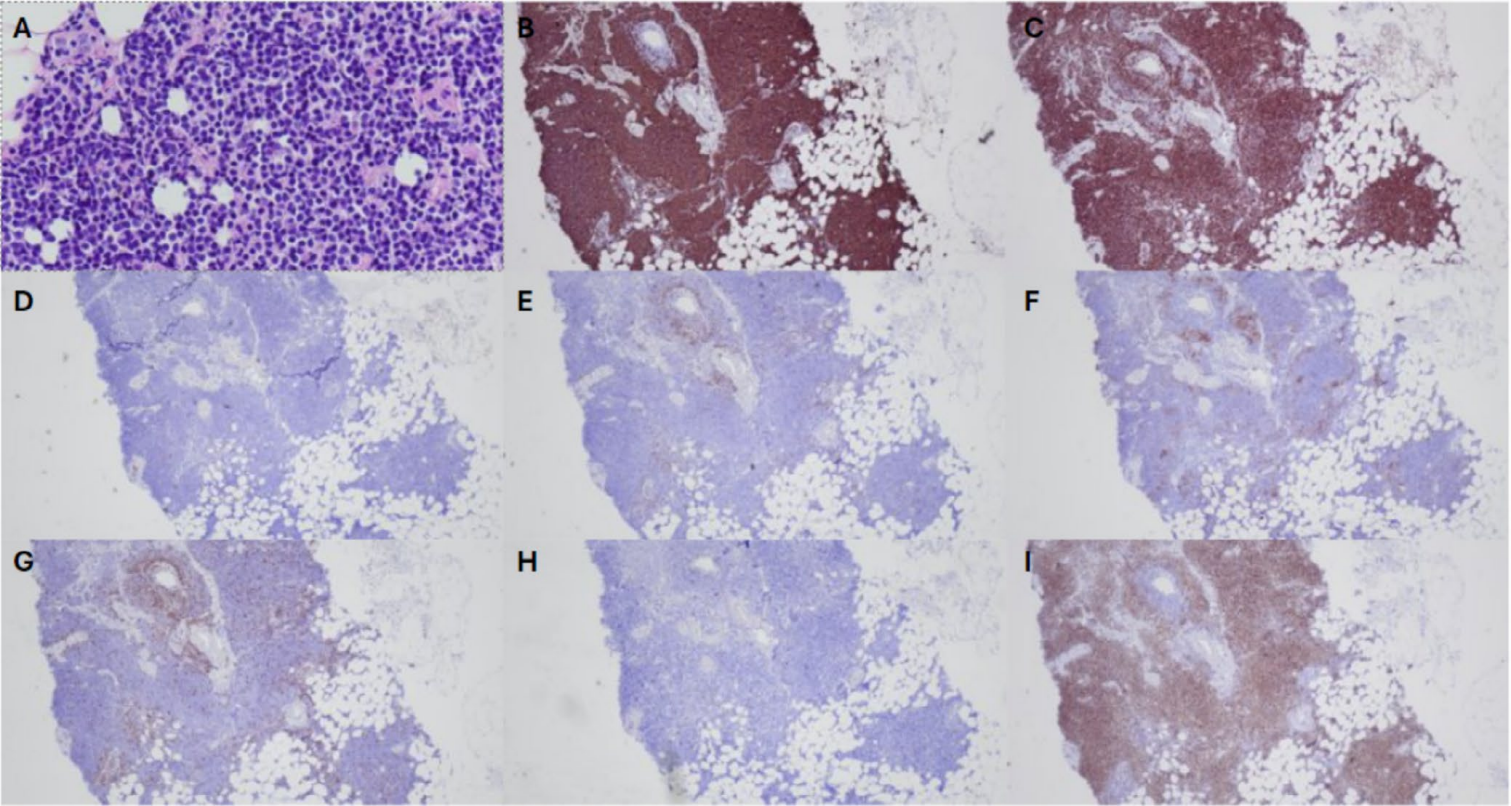
H&E and immunohistochemical staining of biopsy specimen. (A) H&E staining at 400× magnification. (B) Positive staining for CD20 at 40× magnification. (C) Positive staining for Bcl2 at 40× magnification. (D) Negative staining for CD10 at 40× magnification. (E) Negative staining for CD5 at 40× magnification. (F) Negative staining for CD23 at 40× magnification. (G) Negative staining for CD3 at 40× magnification. (H) Negative staining for CyclinD1 at 40× magnification. (I) Positive staining for Pax5 at 40× magnification.

Given the absence of sonographic correlate, stereotactic biopsy was performed, and biopsy results demonstrated features consistent with marginal zone lymphoma, with a dense neoplastic infiltrate of monocytoid cells around blood vessels and very few ducts in the setting of background dense fibrous stroma. The atypical infiltrate stained positive for CD20, PAX5, and BCL2 and negative for CD3, CD5, CD10, BCL6, CyclinD1, and CD23. A few T lymphocytes were highlighted in the background by CD3 and CD5, and CD23 highlighted the follicular dendritic cell meshwork (Figure 2A–I).

Subsequently, a PET‐CT was also performed, demonstrating a low overall disease burden with the known left breast hypermetabolic nodule with moderate FDG‐avidity (Deauville 3) and no evidence of metastatic disease, consistent with Stage IE MALT lymphoma (Figure 1C). Of note, an FDG‐avid tubular opacity in the left upper lobe suggestive of a pulmonary AVM was incidentally found, and a follow‐up CT angiogram of the chest was recommended.

## 4 Conclusions and Follow‐Up

The patient was referred to the hematology‐oncology clinic, at which time the patient was completely asymptomatic. She denied any symptoms of pain, fatigue, night sweats, unintentional weight loss, or fever/chills. The indolent nature of MALT lymphoma and the low but present likelihood of aggressive transformation were reviewed in detail with the patient. As treatment is typically not indicated until systemic symptoms attributable to lymphoma appear, following shared decision‐making, the patient elected to proceed with active surveillance as recommended by the oncology team, with interval imaging every 2–3 years and short interval laboratory follow‐up every 6 months to include complete blood count with differential, lactate dehydrogenase, and complete metabolic panel as well as continuous surveillance of symptoms. Given the incidentally noted pulmonary arteriovenous malformation, CT angiography of the chest was ordered, and Interventional Radiology was consulted, with a recommendation for agitated saline contrast echocardiogram (bubble study) and the potential for further intervention pending the subsequent imaging results.

## 5 Discussion

Primary breast lymphoma is uncommon in its own right, representing fewer than 1% of all breast malignancies. Of these cases, greater than 90% of all PBL cases are of B‐cell origin, with subtypes in declining order of incidence consisting primarily of diffuse large B‐cell lymphoma (approximately 50%–60% of cases), MALT lymphoma, and follicular lymphoma [2, 5]. Presentation of MALT lymphoma is exceptionally rare in the breast, which has been suggested to be due to the relative scarcity of mucosa‐associated lymphoid tissue in the breast [3, 4]. While MALT lymphoma demonstrates a slight female predilection when viewed across all origins, primary breast MALT lymphoma is observed almost exclusively in women [6]. Although the etiology is unclear, an association with chronic inflammation has been shown, particularly Sjogren's syndrome and Hashimoto's thyroiditis or *Helicobacter pylori* in cases of gastric MALT lymphoma. Prior studies have shown evidence that immune mediators such as the CD40/40L signaling pathway, B‐cell receptor engagement, and NF‐kB activation may play roles in the development of MALT lymphoma, with mutations in genes involved in the NF‐kB pathway such as *A20*, *Card11*, *CD79B*, and *Myd88* being most frequently involved [7, 8, 9].

On histology, MALT lymphoma demonstrates diffuse or nodular infiltrate of monocytoid small to medium‐sized cells with slight nuclear irregularity and scant to moderate pale cytoplasm. Reactive follicles are seen in some cases [1]. Additionally, cells classically stain positive for the B‐cell associated antigens CD20 and CD79a, with negative staining for CD5, CD10, and CyclinD1 to rule out small lymphocytic lymphoma, follicular lymphoma, and mantle cell lymphoma, respectively. MALT lymphomas may also be associated with frequent occurrences of a t(11; 18) (q21; q21) translocation, although these are not as common in breast MALT lymphomas [7]. In breast MALT lymphomas, t(11; 18) and t(14; 18)/IGH‐MALT1 have been reported, with rare cases demonstrating trisomies 3, 12, or 18 [5, 7]. Somatic missense mutations in *PIM1* and *cMYC*, missense or frameshift mutations in *p53*, have also been described to occur in up to 20%–40% of gastric and extragastric MALT lymphomas [6, 9].

As there are relatively few cases of MALT lymphoma in the breast, typical imaging findings vary, although presentation may be associated with a patient or physician‐reported palpable region [8, 10]. Of these cases, the most common mammographic findings demonstrate an asymmetry or focal asymmetry without associated calcifications, with sonography demonstrating hypoechoic oval or irregular masses with partially circumscribed or angular margins [6, 10, 11]. However, they may also occasionally be mammographically or sonographically occult, with presentation only visible on other modalities [6, 10]. While there is a paucity of literature regarding the MRI findings, several prior studies reported a variety of findings ranging from T1 isointense and T2 hyperintense irregular enhancing foci with brisk initial enhancement in the wash‐in phase to regional non‐mass enhancement [10, 12].

Most patients are asymptomatic at their diagnosis of MALT lymphoma, notably with an absence of B‐symptoms. They are also generally diagnosed at an early stage (IE or IIE) and demonstrate similar outcomes as compared to localized nodal MZL when evaluating outcomes of gastric origin [7]. As there are significantly fewer cases of MALT lymphoma in the breast, there is a significantly wider range of outcomes in the available literature, although the majority of reported cases appear to show a high overall survival rate with stable disease burden on active surveillance [1, 10].

While treatment is dependent on the tumor location, typical treatment for MALT lymphomas of gastric origin includes systemic chemotherapy and/or radiation therapy, whereas disseminated disease may be treated with any combination of chemotherapy or biologic therapy if indicated. For non‐gastric origins, local radiation therapy has been shown to be the typical primary approach, with an excellent overall survival rate of 96.6% at 5 years [3, 7]. Typically, as these tumors are highly receptive to chemotherapy and radiation, surgical treatment such as wide local excision or mastectomy is rarely indicated [1, 3, 11]. Furthermore, given the generally indolent nature of this diagnosis, treatment may also be deferred entirely even for advanced or disseminated disease unless the patient demonstrates systemic lymphoma‐related symptoms, rapid or increased progression of disease, or concern for end‐organ damage [1, 10]. However, transformation to a clinically aggressive diffuse large B‐cell lymphoma has been reported to occur at a rate of 2%–5%, which would necessitate a corresponding change in treatment plan [13].

## 6 Conclusion

Primary breast MALT lymphoma is an extremely rare entity which frequently presents with variable and subtle imaging findings. The indolent nature of this lymphoma typically allows for a conservative management approach, such as active surveillance, particularly in asymptomatic patients, although this tumor is also highly receptive to chemotherapy and/or radiation if indicated. Our case underscores the importance of maintaining a high index of suspicion in order to obtain the accurate diagnosis for exclusion of more aggressive forms of lymphoma, and utilization of a multidisciplinary approach for optimal patient outcomes. While the prognosis is generally favorable, with high overall survival rates, the potential for transformation to a more aggressive lymphoma necessitates careful monitoring.

## Author Contributions

**Jessica Hui:** visualization, writing – original draft, writing – review and editing. **Amani Bashir:** visualization, writing – original draft, writing – review and editing. **Fabiana Policeni:** writing – review and editing. **Su Kim Hsieh:** conceptualization, writing – original draft, writing – review and editing.

## Consent

Written patient consent has been obtained in accordance with the journal's patient consent policy.

## Conflicts of Interest

The authors declare no conflicts of interest.

## Data Availability

The authors have nothing to report.
